# (A)synchronous Availabilities of N and P Regulate the Activity and Structure of the Microbial Decomposer Community

**DOI:** 10.3389/fmicb.2015.01507

**Published:** 2016-01-06

**Authors:** Nicolas Fanin, Stephan Hättenschwiler, Paola F. Chavez Soria, Nathalie Fromin

**Affiliations:** ^1^Centre d’Ecologie Fonctionnelle et Evolutive, UMR 5175, CNRS, Université de Montpellier, Université Paul-Valéry MontpellierMontpellier, France; ^2^University of Toulouse III Paul SabatierToulouse, France

**Keywords:** decomposition, microbial limitations, multiple element limitation, nutritional constraints, nutrient availability, successive fertilization, temporal variability

## Abstract

Nitrogen (N) and phosphorus (P) availability both control microbial decomposers and litter decomposition. However, these two key nutrients show distinct release patterns from decomposing litter and are unlikely available at the same time in most ecosystems. Little is known about how temporal differences in N and P availability affect decomposers and litter decomposition, which may be particularly critical for tropical rainforests growing on old and nutrient-impoverished soils. Here we used three chemically contrasted leaf litter substrates and cellulose paper as a widely accessible substrate containing no nutrients to test the effects of temporal differences in N and P availability in a microcosm experiment under fully controlled conditions. We measured substrate mass loss, microbial activity (by substrate induced respiration, SIR) as well as microbial community structure (using phospholipid fatty acids, PLFAs) in the litter and the underlying soil throughout the initial stages of decomposition. We generally found a stronger stimulation of substrate mass loss and microbial respiration, especially for cellulose, with simultaneous NP addition compared to a temporally separated N and P addition. However, litter types with a relatively high N to P availability responded more to initial P than N addition and *vice versa*. A third litter species showed no response to fertilization regardless of the sequence of addition, likely due to strong C limitation. Microbial community structure in the litter was strongly influenced by the fertilization sequence. In particular, the fungi to bacteria ratio increased following N addition alone, a shift that was reversed with complementary P addition. Opposite to the litter layer microorganisms, the soil microbial community structure was more strongly influenced by the identity of the decomposing substrate than by fertilization treatments, reinforcing the idea that C availability can strongly constrain decomposer communities. Collectively, our data support the idea that temporal differences in N and P availability are critical for the activity and the structure of microbial decomposer communities. The interplay of N, P, and substrate-specific C availability will strongly determine how nutrient pulses in the environment will affect microbial heterotrophs and the processes they drive.

## Introduction

Nutrient availability often exerts strong limitations on ecological processes in different ecosystem types and across distinct biomes (Vitousek and Howarth, 1991; Elser et al., 2007). Nitrogen (N) and phosphorus (P) play a particularly important role because of their high requirements for building living biomass, and because they determine growth processes and biological activity to a large degree (Sterner and Elser, 2002; Hartman and Richardson, 2013). An important source of N and P, especially for P with ongoing ecosystem and soil development (Walker and Syers, 1976), is dead organic matter of mostly plant origin. Due to the large variability in leaf C: N: P stoichiometry from high to low latitudes (McGroddy et al., 2004), and among phylogenetic groups, life forms or plant species (Hättenschwiler et al., 2008; Yuan and Chen, 2009), microbial decomposers are exposed to substrates of a considerable range in C: N: P (Cleveland and Liptzin, 2007). Recently, Fanin et al. (2013) proposed that C: N: P of leaf litter leachates also control the stoichiometry and structure of the microbial decomposer community. This finding highlights the importance of considering the relevant substrate used by microbial decomposers during the initial stage of decomposition, with leachate C: N: P stoichiometry varying from 169:2:1 to 8280:96:1 among different litter species in the studied tropical rainforest of French Guiana (Fanin et al., 2014). Thus, besides the commonly measured stoichiometry of bulk leaf litter material, the variability of N and P in soluble compounds is likely critical for assessing the effects of any alterations in nutrient availability on microbial communities.

Simultaneous additions of N and P in tropical forests were shown to strongly stimulate litter mass loss and microbial functioning during decomposition (Hobbie and Vitousek, 2000; Reed et al., 2011; Barantal et al., 2012; Fanin et al., 2012). These results support the increasing evidence that ecosystem processes are often co-limited by N and P rather than by N or P alone (Vitousek et al., 2010; Sundqvist et al., 2014). In contrast to the simultaneous application of N and P during fertilization experiments, these two nutrients are unlikely to be available in balanced amounts at the same time in most ecosystems due to their distinct quantities and chemical bonds in dead organic matter and because of their different pathways during biogeochemical cycling. For example, in contrast to N that forms relatively strong chemical bonds with C atoms, ester bonds between P and C atoms are comparatively easier to break, allowing microorganisms to mineralize P from organic molecules with less energy than is required to mineralize N (McGill and Cole, 1981). Thus, although P availability is relatively in excess initially, it may become quickly limiting during the decomposition process. Nutrient availability may also vary throughout the year, with local leaf litter input changing significantly in N: P stoichiometry and in the amount of dissolved organic C between seasons (Wood et al., 2005; Cleveland and Townsend, 2006; Cleveland et al., 2006). Similarly, N or P deposition originating from fossil fuel use, agricultural activity, or from Saharan dust are projected to rise in tropical biomes (Galloway et al., 1994; Okin et al., 2004), but these nutrient inputs are likely to vary substantially over time. For example, P deposition is strongly dependent on Saharan climate and global circulation patterns and has been shown to vary by almost twofold from 20 to 35 kg ha^-1^ year^-1^ (Okin et al., 2004). Despite the large temporal variability in the relative availability of C and nutrients over quite short time spans, its consequences for the structure and function of soil microbial communities remain poorly studied.

Because plant leaf litter represents the most important source of C and nutrients for microbial decomposers, any modification of substrate stoichiometry and nutrient availability can influence extracellular enzyme production (Allison and Vitousek, 2005; Mooshammer et al., 2012; Sinsabaugh et al., 2015), and element-use efficiency of decomposer communities (Manzoni et al., 2012; Mooshammer et al., 2014a). Recently, empirical and theoretical studies showed that shifts in the microbial community structure and in the stoichiometry of the microbial biomass could be one way microbes respond to stoichiometric imbalance with their substrates (Fanin et al., 2013; Kaiser et al., 2014). Because bacteria and fungi present different stoichiometric requirements and constraints (Keiblinger et al., 2010; Strickland and Rousk, 2010), any changes in N and P availability may strongly control the relative proportion of these groups within the community (Krashevska et al., 2010). For instance, Güsewell and Gessner (2009) showed in a microcosm experiment that bacteria were relatively more abundant when decomposition of cellulose was N limited, whereas the relative abundance of fungi increased when cellulose decomposition was P limited. However, whether the timing of N and P availability is critical for the structure and functioning of microbial communities have received only scant attention.

Here we examined how microbial communities respond to successive N and P additions, and whether these responses differ among leaf litter substrates with distinct leachate C: N: P stoichiometry and cellulose as a nutrient free substrate. We specifically addressed the following question: Do successive N and P additions (first N and then P, and *vice-versa*) have different effects compared to simultaneous N and P additions? Although we anticipated that a combined NP addition would stimulate microbial activity more than a temporally separated N and P addition, we hypothesized that this stimulation would be dependent on the substrate quality. Specifically, we anticipated that the effects of sequential nutrient supply would depend on the N: P ratio of the soluble fraction of the litter substrates: (i) litter that is relatively rich in soluble N or P would respond stronger when P or N is added first, respectively, (ii) litter that is relatively well balanced in soluble N and P would not show a particular response to either N or P added first, and (iii) cellulose as a nutrient-free substrate would display the strongest effects when both nutrients are added simultaneously (Hypothesis 1). Because these two nutrients show distinct release patterns from decomposing litter, we hypothesized that the order in the relative N- and P-availability would determine microbial process rates, with: (i) P added first and then N resulting in the weakest effect because P is relatively available early during the decomposition process, (ii) N added first and then P showing an intermediate effect as P is relatively more limiting later during the decomposition process, and (iii) a simultaneous NP addition having the strongest effects because of synergistic responses when both elements are available in excess (Hypothesis 2). Finally, as a result of different nutrient requirements between fast-growing bacteria and more slowly growing fungi (Güsewell and Gessner, 2009; Keiblinger et al., 2010), we hypothesized that the relative abundance of these different microbial groups would be altered in a predictable way by N and P additions, with: (i) P added first and then N leading first to an increased proportion of bacteria that decreases again when N is added (ii) N added first and then P increasing the relative proportion of fungi in a first time which decreases again once P is added, (iii) simultaneous NP addition stimulating both fungal and bacterial biomasses with no net change in their relative abundance (Hypothesis 3). We tested our hypotheses with a laboratory microcosm experiment by measuring litter mass loss, microbial activity (substrate-induced respiration, SIR), and microbial community structure (phospholipid fatty acids, PLFAs) in the litter and the underlying soil during the initial stage of decomposition.

## Materials and Methods

### Soil and Litter Material

The soil used for our experiment was collected from the topsoil (0–10 cm) in the Amazonian rainforest, at Paracou, French Guiana (5°15′N, 52°53′W). It is classified as a nutrient-poor acrisol developed over a Precambrian metamorphic formation called the Bonidoro series with a mean pH of 4.4 in the top soil and a soil texture with 20% clay, 6% silt and 74% sand, with a total C of 22.1 g kg^-1^, a total N of 1.5 g kg^-1^ soil and a total P of 0.10 g kg^-1^ soil (see Fanin et al., 2011 for more details). The soil was dried at 25°C, sieved at 2 mm, homogenized, and stored dry until use.

Air-dried leaf litter of three different tree species occurring at our research site [*Goupia glabra* (Aublet), *Simarouba amara* (Aublet) and *Vochysia tomentosa* (G. Mey.)] as well as cellulose paper (filter paper qualitative 410, VWR, Fontenay-sous-Bois, France) were used as substrates for decomposition. In addition to leaf litter as a naturally occurring substrate for microbial growth, we used cellulose as a widely accessible C source for a large part of the heterotrophic microbial community that contains no other elements than C, O and H. Cellulose served as an extreme end point along the stoichiometric gradient covered by the three litter species and also to add a single C compound compared to the litter species composed of diverse and distinct C compounds (**Table 1**). The three litter species were selected based on their distinct C: N: P stoichiometry of the soluble fraction (**Table 1**) that has previously been shown to have a major impact on litter and soil microbial communities (Fanin et al., 2013, 2014). The three species also showed contrasting responses to combined N and P fertilization in a previous decomposition experiment in the field, with a very small effect on *V. tomentosa*, an intermediate effect on *S. amara*, and a strong effect on *G. glabra* (Fanin et al., 2012).

**Table 1:** Chemical characteristics of litter species (mean ± SD) used for the microcosm incubations.

Litter traits	Cellulose	Goupia glabra	Simarouba amara	Vochysia tomentosa
**Bulk material**				
C (mg g^-1^)	445	497 ± 2	491 ± 1	429 ± 4
N (mg g^-1^)	0	12, 1 ± 1, 3	11, 1 ± 0, 7	8, 7 ± 0, 4
P (mg g^-1^)	0	0, 33 ± 0, 04	0, 32 ± 0, 02	0, 29 ± 0, 01
C: N	∞	41, 1 ± 4, 1	44, 2 ± 2, 9	49, 3 ± 2, 9
C: P	∞	1507 ± 168	1534 ± 111	1479 ± 87
N: P	–	36, 7 ± 1, 5	34, 7 ± 0, 7	30 ± 2, 9
C: N: P	–	1507:37:1	1534:35:1	1479:30:1
**Soluble fraction**				
solC (mg g^-1^)	–	19, 3 ± 2, 5	10, 7 ± 0, 6	7, 5 ± 0, 3
solN (mg g^-1^)	–	54, 2 ± 3, 5	125, 0 ± 8, 3	86, 4 ± 3, 9
solP (mg g^-1^)	–	46, 1	36, 4	0, 9
solC: N	∞	355, 5 ± 25, 8	85, 8 ± 5, 3	86, 3 ± 2, 4
solC: P	∞	419 ± 54	294 ± 15, 9	8280 ± 297
solN: P	–	1, 2 ± 0, 1	3, 4 ± 0, 2	96, 0 ± 4, 4
solC: N: P	–	419:1:1	294:3:1	8280:96:1
***Carbon forms***				
Water soluble compounds (%DM)	–	36.6 ± 0.4	45.4 ± 0.4	34.6 ± 1.1
Hemicellulose (%DM)	–	16.2 ± 0.7	11.7 ± 0.2	20.1 ± 1.1
Cellulose (%DM)	100	18.8 ± 0.3	20.0 ± 0.3	19.7 ± 0.4
Lignin (%DM)	–	28.4 ± 0.8	22.8 ± 0.7	25.6 ± 0.4
Total phenolic (%DM)	–	1.1 ± 0.2	4.4 ± 0.2	0.6 ± 0.1
Soluble phenolic (%DM)	–	2.8 ± 0.3	11.0 ± 0.8	4.4 ± 0.4
Tannin (%DM)	–	0.6 ± 0.1	6.3 ± 0.3	3.9 ± 0.3

Freshly fallen leaf litter was collected with 25 m × 25 m large litter traps installed in mono-specific stands of an experimental plantation adjacent to the Paracou natural forest station (Roy et al., 2005), air-dried immediately upon collection, pooled across sampling dates, and stored dry. Only intact leaves without signs of herbivory, galls or fungal attack were kept (see Fanin et al., 2012 for further details on leaf litter collection).

### Experimental Design

For testing the impact of the changing relative availability of N and P through time on decomposition and microbial decomposer communities, we defined three different fertilization treatments. The first treatment consisted in the combined addition of N and P at the same time (denoted as ‘*NP*’ in the following). In the second treatment we applied first N and later P (denoted as ‘*Np*’ in the following), and in the third treatment we applied first P and later N (denoted as ‘*Pn*’ in the following). A control treatment without any fertilizer application was also included. Nutrients were added either at the beginning of the experiment (d_0_), and/or 36 days after the start of experiment (d_36_) depending on the treatment. We added N as KNO_3_ and NH_4_NO_3_, and P as KH_2_PO_4_ at the same total quantities of N and P as used in a fertilization experiment in the field at the research site in Paracou (Barantal et al., 2012), i.e., 0.055 g KNO_3_ and 0.069 g NH_4_NO_3_ per microcosm, and 0.074 g KH_2_PO_4_ per microcosm. We used KNO_3_ in addition to NH_4_NO_3_ to keep the added K constant between the N and P treatments. The total amounts of added N and P correspond to 130 kg N ha^-1^ y^-1^, and 69 kg ha^-1^ y^-1^, respectively, which is comparable to the quantities used in other fertilization experiments in tropical forests (see Hobbie and Vitousek, 2000; Cleveland et al., 2006; Kaspari et al., 2008).

The microcosms consisted of 30 cm wide × 15 cm long × 10 cm tall plastic boxes that were covered with a plastic cap. Each microcosm received 80 g of homogenized air dry soil and 4 g (oven dried-corrected weight) of air dry leaf litter material or cellulose paper that was placed on the top of the soil. Leaf litter and cellulose paper were cut in 1 cm × 1 cm squares in order to homogenize leaf margin: total surface area ratio across all substrates. For each microcosm, we soaked the 4 g-substrate aliquotes in 40 mL of distilled water (for the *control* treatment) or in 40 mL of the corresponding fertilizer solution (for the *NP, Np*, and *Pn* treatments) while shaking for 24 h in order to ensure complete and homogeneous rewetting of the substrates before their addition to the microcosms. Along with the substrates we also added the soaking solution to each individual microcosm at d_0_, which corresponded to the volume of water needed to reach 80% of water holding capacity of the soil in the microcosm. For the second fertilization event at d_36_, 10 mL distilled water or fertilizer solutions (four times more concentrated than at d_0_ in order to add the same quantity of nutrients while keeping soil humidity close to 80% of water holding capacity) were added to all microcosms of the respective treatments. During the entire experimental duration of 74 days, the microcosms were kept at constant temperature of 30°C and under water saturated atmosphere, and microcosms were sprinkled to their original weight with distilled water every 4 days in order to keep soil and litter humidity close to constant.

We constructed a total of 12 microcosms for each combination of litter material (*G. glabra, S. amara, V. tomentosa* and cellulose paper) and fertilization treatment (control, *NP, Np*, and *Pn*), resulting in a total of 192 microcosms. Three microcosms for each treatment combination were harvested after 18 (d_18_) and 36 (d_36_) days, just before the second fertilization event, and after 54 (d_54_) and 74 days of incubation (d_74_).

### Response Variables

At each sampling date and for each microcosm, the remaining litter material was carefully retrieved, cleaned from soil particles, and weighed for fresh remaining mass. A litter subsample was dried for 48 h at 65°C for dry mass conversion and the calculation of litter mass loss in each microcosm. Additional subsamples of litter and of homogenized soil were immediately frozen after pooling the three replicates of each treatment for further PLFA analyses (*n* = 1 for each litter × treatment combination). The remaining material was air-dried at 25°C.

Substrate induced respiration (SIR), as an integrative indicator of the overall microbial activity (Nannipieri et al., 2003), was determined for soil and litter material as previously described (Fanin et al., 2012). Briefly, 10 g of air dry soil were placed in a sealed 150 mL flask and received a solution of glucose in order to reach 80% of the soil water holding capacity and to add 1.5 mg C g^-1^ of soil. For litter materials, 2 g of air dry litter was incubated in the same way, but adding glucose solution to reach 20 mg C g^-1^ of litter. The flasks were incubated at 25°C for 6 h, a time span that is considered as short enough to avoid *de novo* enzyme synthesis. Two hundred micro liter headspace air samples were analyzed after 2 and 6 h for CO_2_ concentration using a gas chromatograph with catharometric detection (Varian CP-4900, Walnut Creek, CA, USA). The amount of CO_2_ released during the 4 h time lapse was used to calculate SIR rate expressed in μg C-CO_2_ g^-1^ soil or litter h^-1^.

Microbial community structure was determined by analyzing group-specific PLFAs from a representative subsample of 1 and 10 g fresh weight frozen litter material and soil, respectively, using the protocol described in Fanin et al. (2014). Branched and saturated, mono-unsaturated and cyclopropyl PLFAs i15:0, a15:0, i16:0, i17:0, a17:0 16:1ω7c, cy17:0, 18:1ω7c, cy19:0 were used to characterize bacteria while the 18:1ω9, 18:2ω6,9 PLFAs were used as biomarkers of fungi (e.g., Frostegård and Bååth, 1996; Zelles, 1999; Bååth, 2003).

### Data Analysis

We analyzed litter mass loss, litter SIR, and soil SIR at the different sampling dates using repeated measures ANOVAs in which litter material, fertilizer treatment, and sampling date were treated as fixed effects and were permitted to interact. Microcosm identity was included as a random factor to account for the repeated sampling over time. For each litter material, we also compared the differences between treatments for each sampling date, followed by a *post hoc* test of Tukey-HSD (α = 0.05). Using Bray–Curtis dissimilarity matrices on the selected 23 PLFAs markers, we used permutational multivariate ANOVAs (PERMANOVA) to test the effects of treatment, litter material and sampling date on microbial community structure. Following repeated measure ANOVA, we used the % sum of square as a comparative measure of % variation explained by the different factors (substrate identity, fertilization treatment, sampling date and their interactions). We run non-metric multidimensional scaling (NMDS) with vector fitting, based on multiple linear regressions using the coordinates on the first two principal axes as the explanatory variables, and the variable of interest (e.g., relative abundance of microbial groups) as the dependent variable. Finally, to visualize the effect of N and P availability on microbial community structure, we compared the fungi to bacteria ratio (fungi:bacteria) across all substrate types as a function of the different fertilization treatments at d_36_ and d_74_. Cellulose was excluded from this analysis because of the large differences in microbial community responses compared to the three other species. All statistical tests were performed with the R software (version 2.11.1).

## Results

### Fertilization and Substrate Identity Effects on Litter Mass Loss

**FIGURE 1:**
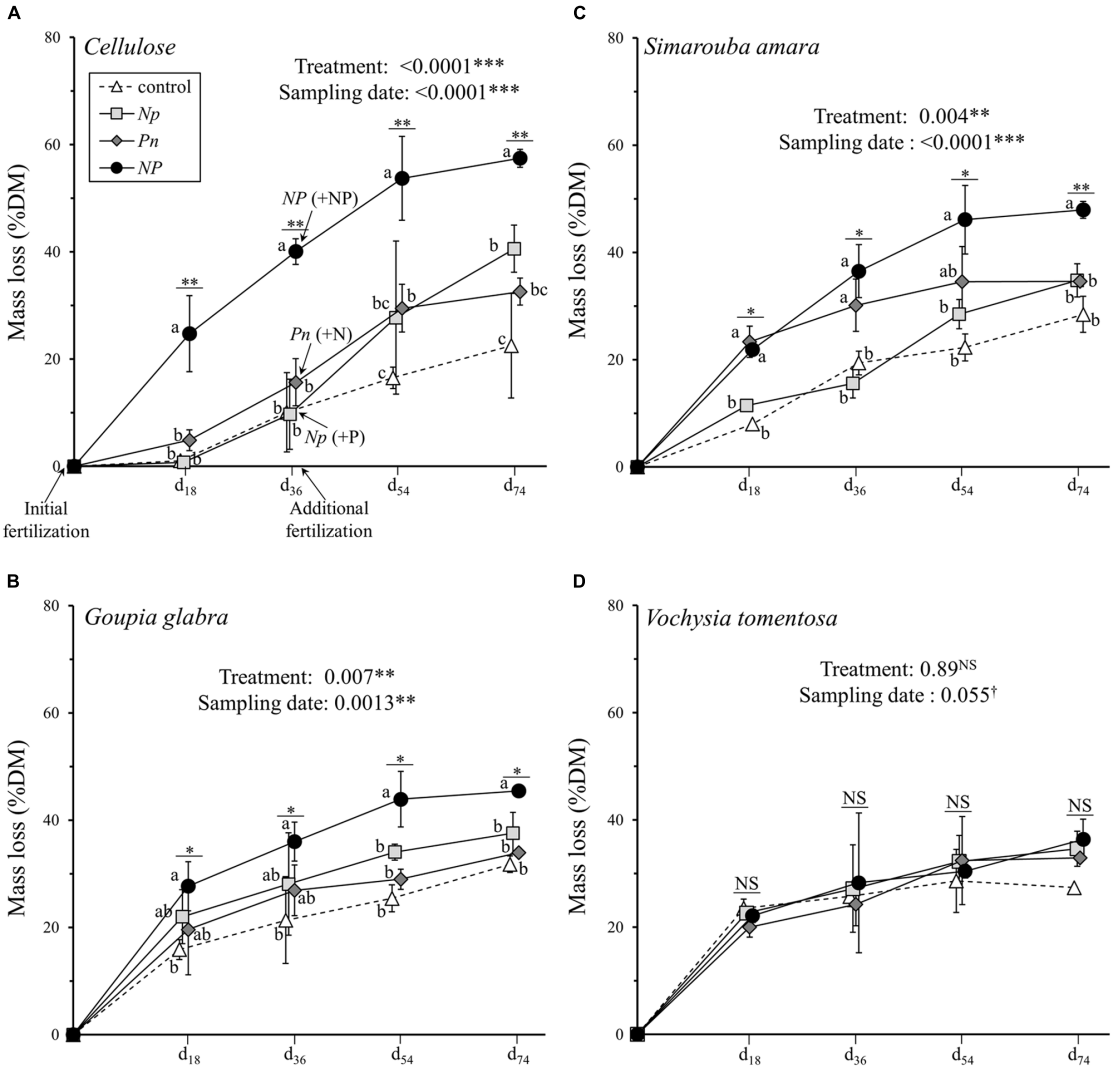
**Mass loss of the four different substrates (mean ± SD) as a function of sampling date. (A)** cellulose, **(B)** *Goupia glabra*, **(C)** *Simarouba amara*, **(D)** *Vochysia tomentosa*. Fertilization treatments are shown with distinct symbols and curves (control: open triangle, dotted line; *NP*: black circles, solid line; *Np*: light gray square, solid line; *Pn*: dark gray diamond, solid line). Microcosms received the first dose of fertilizer (N, P, or NP) at d_0_, and the second dose of fertilizers (i.e., P, N, or NP respectively) just after d_36_ sampling. Stars indicate significant differences in the mass loss for each date considered separately, followed by a *post hoc* Tukey-HSD test (α = 0.05), with different letters indicating contrasted effects among the different fertilization treatments.

Sampling date (28.4% of the variation explained), fertilization treatment (16.1%), and substrate identity (6.1%) all had significant effects on substrate mass loss (**Table 2A**). The interaction between substrate identity and fertilization treatment also explained 11.3% of the observed variation in substrate mass loss, i.e., a higher amount of variation than the substrate identity alone. Of all substrates, cellulose showed the largest variation in mass loss among the fertilization treatments, ranging from 22.5% of initial mass lost on average in the control treatment to 57.4% of initial mass lost in the *NP* treatment at the final harvest after 74 days of decomposition (**Figure 1A**). The other two fertilization treatments where N and P were added successively showed intermediate final mass loss of 32.6% for *Pn* and 40.6% for *Np*. Despite the marginally significant interaction between sampling date and fertilization treatment (**Table 2A**), cellulose mass loss in response to N or P addition (as the first phase of the *Np* or *Pn* treatments, respectively) did not differ from the control treatment during the first 36 days (**Figure 1A**). After the additional fertilization event (i.e., when the second nutrient was added), cellulose mass loss was faster in both treatments (*Np* and *Pn*) compared to the control treatment, although still remaining lower as compared to cellulose fertilized with both nutrients simultaneously. Opposite to cellulose, *V. tomentosa* leaf litter was not affected by any of the fertilization treatments (**Figure 1D**). *G. glabra* and *S. amara* leaf litters showed intermediate responses to the fertilization treatments compared to cellulose and *V. tomentosa* leaf litter. After 74 days, mass loss in the *NP* treatment was 13.7 and 19.4% higher than in the control for *G. glabra* and *S. amara*, respectively (**Figures 1B,C**). Adding P first clearly enhanced decomposition of *S. amara* but not that of *G. glabra* during the first part of the experiment. In both, *Np* and *Pn* treatments, the addition of the second nutrient at d_36_ influenced further decomposition only little, and the differences to mass loss in the control treatment got overall rather smaller with ongoing experimental duration.

**Table 2:** Repeated measures ANOVA evaluating the role of substrate identity, treatment and sampling dates: (A) Litter mass loss, (B) SIR Litter, (C) SIR Soil.

	df	%SS	SS	MS	F.model	*p-value*
**(A) Litter mass loss**						
Substrate identity	3	6.1	1627	542.3	7.6	<0.0001
Fertilization treatment	3	16.1	4324	1441.4	20.2	<0.0001
Sampling date	3	28.4	7625	2541.6	35.6	<0.0001
Substrate identity × Fertilization treatment	9	11.3	3038	337.6	4.7	<0.0001
Fertilization treatment × Sampling date	9	4.3	1146	127.3	1.8	0.078
Substrate identity × Sampling date	9	1.5	405	44.9	0.6	0.77
Residuals	122	32.4	8710	71.4		
**(B) SIR Litter**						
Substrate identity	3	31.8	9.5	3.2	56.9	<0.0001
Fertilization treatment	3	10.6	3.2	1.1	18.9	<0.0001
Sampling date	3	5.9	1.8	0.6	10.5	< 0.0001
Substrate identity × Fertilization treatment	9	5.2	1.6	0.2	3.1	0.002
Fertilization treatment × Sampling date	9	8.5	2.5	0.3	5.1	< 0.0001
Substrate identity × Sampling date	9	10.8	3.2	0.4	6.5	< 0.0001
Residuals	146	27.2	8.1	0.1		
**(C) SIR Litter**						
Substrate identity	3	31.3	107.8	35.9	59.4	<0.0001
Fertilization treatment	3	11.3	39.0	13.0	21.5	<0.0001
Sampling date	3	5.6	19.3	6.4	10.6	<0.0001
Substrate identity × Fertilization treatment	9	7.6	26.4	2.9	4.8	<0.0001
Fertilization treatment × Sampling date	9	7.6	26.2	2.9	4.8	<0.0001
Substrate identity × Sampling date	9	11.0	37.9	4.2	7.0	<0.0001
Residuals	146	25.6	88.4	0.6		

*Repeated measures ANOVAs were performed to examine the relative influence of litter material, fertilization treatment, and time, as well as interactions between the three explanatory variables in accounting for the variation in microbial activity over the course of the incubation experiment.*

### Fertilization and Substrate Identity Effects on SIR

**FIGURE 2:**
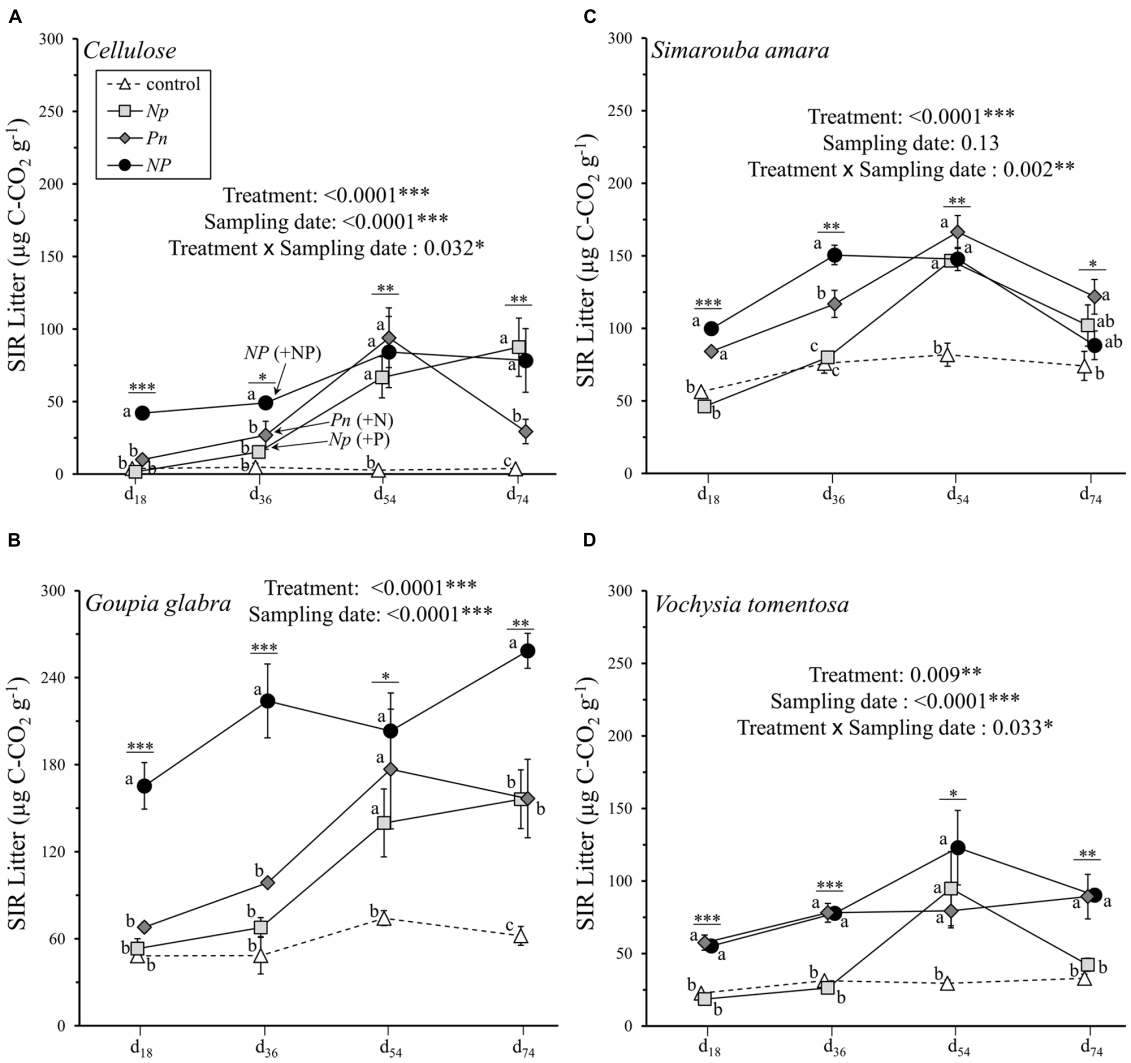
**Litter SIR rates of the four different substrates (mean ± SD) as a function of sampling date. (A)** cellulose, **(B)** *Goupia glabra*, **(C)** *Simarouba amara*, **(D)** *Vochysia tomentosa*. Fertilization treatments are shown with distinct symbols and curves (control: open triangle, dotted line; *NP*: black circles, solid line; *Np*: light gray square, solid line; *Pn*: dark gray diamond, solid line). Stars indicate significant differences in the mass loss for each date considered separately, followed by a *post hoc* Tukey-HSD test (α = 0.05), with different letters indicating contrasted effects among the different fertilization treatments.

Substrate induced respiration in decomposing material varied between 1.8 and 258.6 μg C-CO_2_ g^-1^ across all substrates and all sampling dates (**Figure 2**). In contrast to litter mass loss, litter SIR was primarily influenced by substrate identity and its interaction with sampling date, explaining 31.8 and 10.8% of the overall variation, respectively (**Table 2B**). A significant, though lower amount of variation in litter SIR was explained by fertilization treatment (10.6%) and its interaction with sampling date (8.5%). Prior to the second fertilization event, SIR rates were highest in cellulose in the NP treatment (49.2 μg C-CO_2_ g^-1^ at d_36_), while SIR rates following the supply of N or P alone did not differ from the control treatment between 0 and 36 days (**Figure 2A**). When the second nutrient was added, SIR increased to 94.0 and 66.8 μg C-CO_2_ g^-1^ at d_54_ in the *Pn* and *Np* treatments, respectively, reaching values similar to those when cellulose was fertilized with N and P at the same time (*NP* treatment). Without any nutrient addition (control treatment), SIR rates on cellulose were null at all of the sampling dates. Similar to cellulose, *G. glabra* showed a substantial stimulation of litter SIR following the first fertilization event when N and P were added together, reaching 223.9 μg C-CO_2_ g^-1^ at d_36_ on average (**Figure 2B**). After the second fertilization, SIR rates of *G. glabra* in *Pn* and *Np* treatments were higher than in the control and did not differ to those observed in *NP*. But on d_74_ the stimulation of SIR was highest in *NP* and intermediate in *Np* and *Pn* as compared to the control. Similar to cellulose and *G. glabra*, SIR rates of *V. tomentosa* and *S. amara* leaf litter were also higher at d_36_ with a combined *NP* fertilization than with N supplied alone, and also with higher SIR rate when P was added compared to N (**Figures 2C,D**). Following the second fertilization, and as observed for cellulose and *G. glabra*, SIR rates at d54 increased following P supply in the *Np* treatments for both litter species, and with N supply in the *Pn* treatment on *S. amara*, and reached similar values to those observed when both resources were added simultaneously (*NP* treatment). But at the final d_74_ sampling, the stimulation of SIR rates of *V. tomentosa* following *Np* did not persist.

**FIGURE 3:**
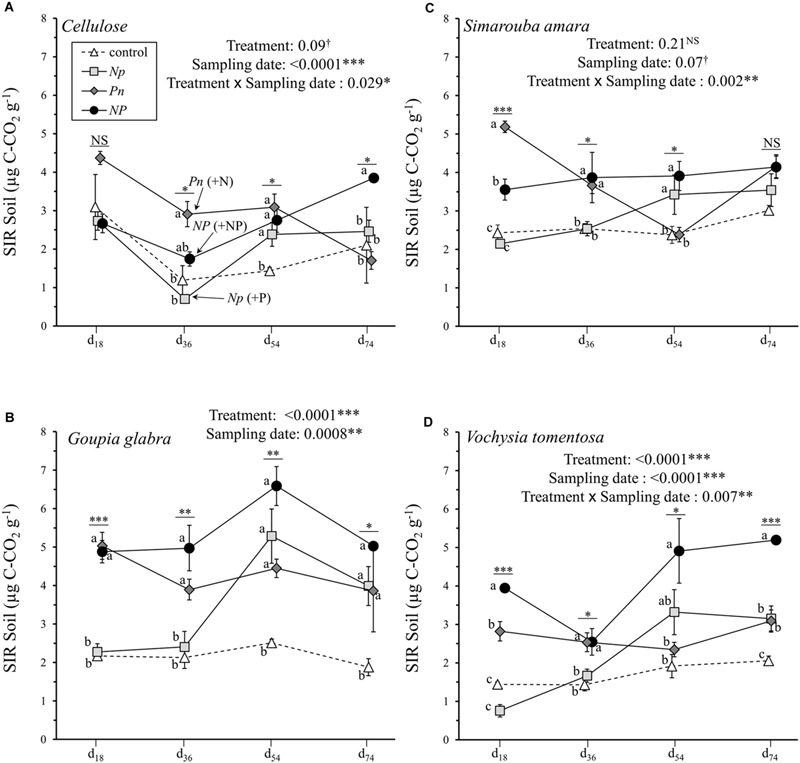
**Soil SIR rates of the four different substrates (mean ± SD) as a function of sampling date. (A)** cellulose, **(B)** *Goupia glabra*, **(C)** *Simarouba amara*, **(D)** *Vochysia tomentosa.* Fertilization treatments are shown with distinct symbols and curves (control: open triangle, dotted line; *NP*: black circles, solid line; *Np*: light gray square, solid line; *Pn*: dark gray diamond, solid line). Stars indicate significant differences in the mass loss for each date considered separately, followed by a *post hoc* Tukey-HSD test (α = 0.05), with different letters indicating contrasted effects among the different fertilization treatments.

Substrate induced respiration measured in the soil underneath the decomposing substrates was generally distinctively lower than that measured in decomposing material, varying between 0.70 and 6.59 μg C-CO_2_ g^-1^ soil (**Figure 3**). The hierarchy of factors explaining soil SIR over the incubation period were similar to those observed for litter SIR, with a large part of the variation explained by substrate identity (31.3%), fertilization treatment (11.3%) and sampling date (5.6%) as well as interactions between factors (**Table 2C**). Overall, *NP* addition and *P* only addition treatments showed higher soil SIR rates at d_36_ compared to N only addition or control treatment, except for cellulose where *NP* did not differ from the control (**Figure 3A**). The addition of N in the *Pn* treatment did not show any influence on the soil SIR rates under *V. tomentosa, G. glabra* and cellulose, and even a trend for a negative effect under *S. amara* (**Figure 3C**). Opposite to this pattern, the addition of P in the *Np* treatment stimulated soil SIR rates, especially under *G. glabra* with an increase from 2.40 to 5.28 μg C-CO_2_ g^-1^ between d_36_ and d_54_ (**Figure 3B**). Differences between treatments were, however, smaller at the end of the incubation, except for *V. tomentosa* with a higher soil SIR rate of 5.19 in *NP* compared to 2.05 in the control (**Figure 3D**).

### Fertilization and Substrate Identity Effects on the Microbial Community Structure

**FIGURE 4:**
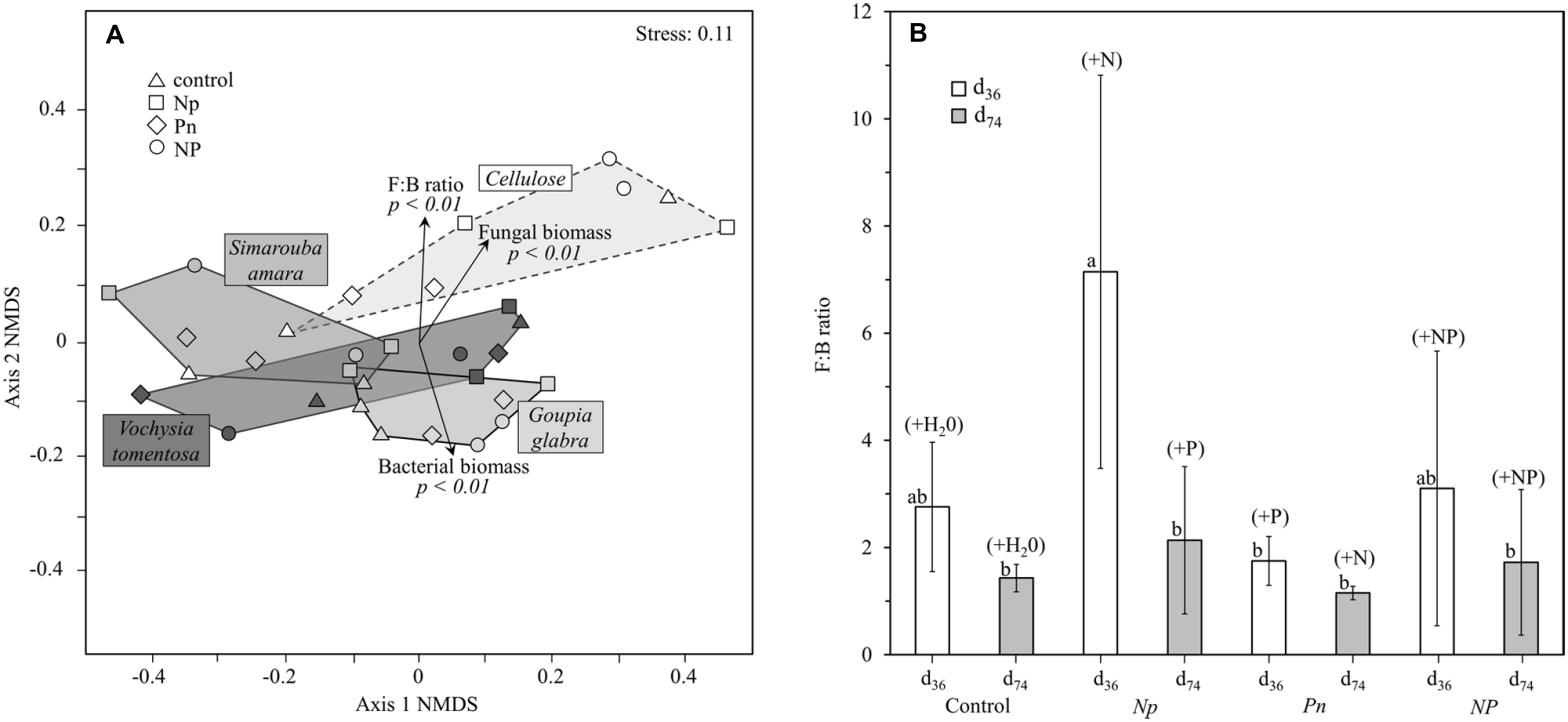
**Microbial community structure in leaf litter and cellulose: **(A)** NMDS ordination of microbial community composition by substrate identity, **(B)** fungi:bacteria ratio across all substrate types (except cellulose) as a function of the different fertilization treatments at d_36_ and d_74_.** NMDS was based on Bray–Curtis distances of 23 PLFA markers. With increasing distance between two points the community structure becomes more dissimilar. Vectors represent the proportion of the main microbial groups (bacteria, fungi) and their ratio (fungi:bacteria), pointing in the direction of higher abundances or a higher fungi:bacteria ratio.

The structure of the microbial community (i.e., relative abundance of the 23 group-specific PLFA markers) differed strongly among the decomposing substrates, with contrasted NMDS plots mostly driven by cellulose that showed a distinct PLFA pattern compared to leaf litter along the second NMDS axis (**Figure 4A**). Such differences in microbial community structure between leaf litter and cellulose were clearly related to lower fungal lipid markers in leaf litter than in cellulose that translated into lower litter fungi:bacteria (see Supplementary Table S1 for more details). PERMANOVA to test for the effects of the different treatments on PLFA markers revealed that substrate identity accounted for 42.1% of the overall variation of the microbial community structure, which is roughly as much as that accounted for by all the remaining factors and interactions (**Table 3A**). To a lower extent, the structure of the microbial community in the decomposing substrates also differed across the fertilization treatments and their interactions with sampling date and substrate identity (6.4, 20.1, and 13.8% of explained variation, respectively). When omitting the cellulose substrate from the comparison, the effects of fertilization treatments were particularly apparent on the leaf litter fungi:bacteria (**Figure 4B**). The addition of N only strongly decreased the relative proportion of bacterial lipid markers by 49% whereas the addition of P only increased it by 57% on average. Such change resulted in contrasting fungi:bacteria of 7.1 (with N only) or 1.7 (with P only), respectively, compared to 2.8 in the control treatment. After complementary P addition in the *Np* treatment or N addition in the *Pn* treatment, the fungi:bacteria shifted to almost similar values. No significant difference was observed among the fertilization treatments at d_74_.

**Table 3:** Permutational multivariate ANOVA evaluating the role of substrate identity, fertilization treatment and sampling dates on microbial community structure in (A) litter, and (B) soil.

	df	SS	MS	F.model	*p-value*	*r^2^*
**(A) Litter community structure**						
Substrate identity	3	0.84	0.28	13.0	0.001	0.421
Fertilization treatment	3	0.13	0.04	2.0	0.002	0.064
Sampling date	1	0.08	0.08	3.9	0.035	0.042
Substrate identity × Fertilization treatment	9	0.27	0.03	1.4	0.002	0.138
Fertilization treatment × Sampling date	3	0.07	0.02	1.1	0.001	0.201
Substrate identity × Sampling date	3	0.40	0.13	6.2	0.373	NS
Residuals	9	0.19	0.02			0.097
**(B) Soil community structure**						
Substrate identity	3	0.14	0.05	1.9	0.31	NS
Fertilization treatment	3	0.05	0.0.2	0.7	0.29	NS
Sampling date	1	0.18	0.18	7.2	0.002	0.187
Substrate identity × Fertilization treatment	9	0.17	0.02	0.8	0.59	NS
Fertilization treatment × Sampling date	3	0.10	0.03	1.4	0.032	0.107
Substrate identity × Sampling date	3	0.09	0.03	1.2	0.33	NS
Residuals	9	0.22	0.02			0.235

*Permutational multivariate ANOVAs were performed to examine the relative influence of litter material, fertilization treatment, and time, as well as interactions between the three explanatory variables in accounting for the variation in microbial community composition over the course of the incubation experiment.*

**FIGURE 5:**
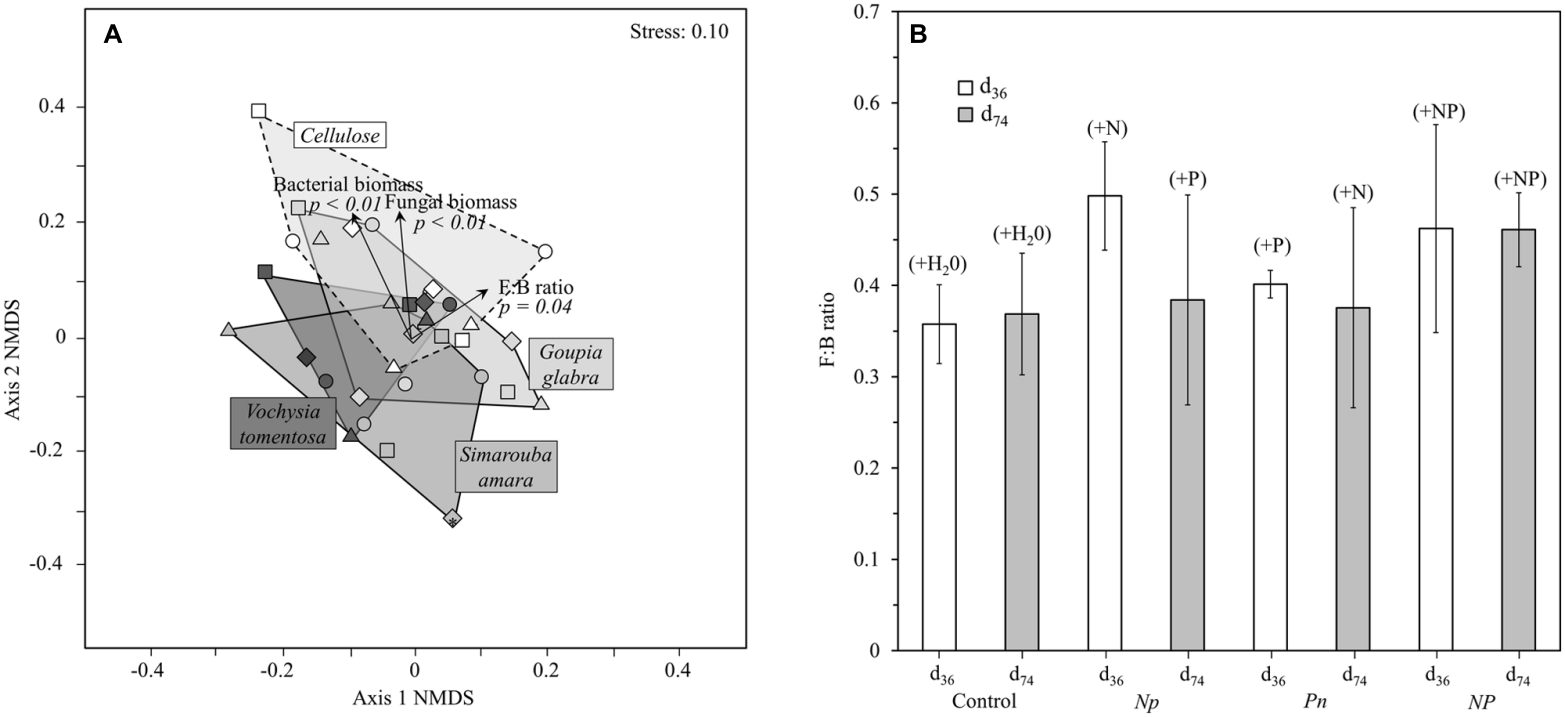
**Microbial community structure in the soil: **(A)** NMDS ordination of microbial community composition by substrate, **(B)** fungi:bacteria ratio across all substrate types (except cellulose) as a function of the different treatments at d_36_ and d_74_.** See **Figure 4** for further details.

Microbial community structure in the soil was affected by different factors compared to that in decomposing substrates (**Table 3B**). Neither substrate identity nor fertilization treatment and their interactions explained the PLFA-based microbial community structure in the soil. In contrast, sampling date and its interaction with fertilization treatment accounted for 18.7 and 10.7% of the total variation observed in soil microbial community structure, respectively. We did not find any clear effect of the substrate identity on the soil community structure in the NMDS plot (**Figure 5A**). By contrast to the litter microbial community and even after omitting the cellulose substrate, we did not find any clear effects of fertilization or sampling date on the PLFA-based soil microbial community structure (**Figure 5B**).

## Discussion

In line with the increasing number of studies demonstrating that the combined NP additions increase ecosystem processes more than P or N added singly (e.g., Vitousek et al., 2010), we found an overall stronger stimulation of substrate mass loss and litter microbial activity with a simultaneous NP supply compared to a temporally separated N and P addition. The fact that N and P together constrain the activity of microbial communities was particularly clear for litter SIR rates that on average varied sevenfold between the control and the *NP* treatments throughout the incubation period (**Figure 2**). These results are in line with previous studies showing that the decomposition of labile C substrates (Nottingham et al., 2012, 2015) or more complex leaf litter (Hobbie and Vitousek, 2000; Barantal et al., 2012; Fanin et al., 2012) were predominantly limited by concomitant NP additions. Because the stoichiometry of N and P supply and demand is generally in close balance in most terrestrial ecosystems (Vitousek et al., 2010), N is rarely available in great excess relative to P, and a slight addition of P can rapidly generate a N limitation or *vice-versa*: N and P limitation may thus alternate in multiple incremental steps, ultimately producing a synergistic effect when both nutrients are added together (Davidson and Howarth, 2007). In contrast to the ‘Liebig world’ view, which states that the nutrient in the shortest supply will be limiting, we demonstrate here that both elements can constrain litter decomposition and are required by microbial communities for maintaining their activity.

Beyond evaluating the effects of simultaneous NP additions, we addressed how the temporal variability in N and P availability affects litter decomposition and the activity of decomposer organisms. As expected following our first hypothesis, the responses of microbial parameters to a temporally separated N and P addition varied strongly among litter substrates (**Table 2**). We found that P supply showed a higher effect on SIR rates for the litter species with the highest soluble N content (i.e., *S. amara* and *V. tomentosa*), whereas the respective effects of N or P additions were similar when these two elements are relatively well balanced in litter leachates (i.e., *G. glabra*), or when they are completely absent (i.e., cellulose) (**Figure 2**). These results suggest that the relative imbalance between N and P in litter leachates can predict relatively well which of these two elements will be limiting during the initial phase of decomposition and may thus be used as a general indicator of nutritional constraints regulating the microbial activity. In other words, because the timing of nutrient availability in a given ecosystem can vary, depending for example on seasonal differences such as dry *versus* wet season in tropical forests (e.g., Turner et al., 2015), considering N and P in the soluble fraction of plant leaf litter may contribute to the understanding of temporal variability in soil processes.

Interestingly, after adding the second nutrient in the middle of the course of the experiment (i.e., N to the *Pn* treatment, and P to the *Np* treatment), SIR generally increased to similar rates as those observed in the *NP* treatment (**Figure 2**). In contrast to our second hypothesis, the similar responses of microbial processes regardless of whether N or P was added first suggest that the order in relative N- and P-availability does not determine final microbial process rates, and confirms that litter decomposition and microbial activity are limited by multiple elements in interaction (Townsend et al., 2011; Fanin et al., 2015). In parallel, we observed a lower relative abundance of bacteria compared to fungi in response to N fertilization, in line with our expectation of enhanced fungal biomass following N additions (Koranda et al., 2014). Such a shift in the fungi to bacteria ratio was reversed following further addition of P later in the *Np* treatment (**Figure 4**), suggesting that P is a more critical element for the fast-growing bacterial community compared to the relatively slower growing fungal community (Güsewell and Gessner, 2009). Thus, although the PLFA results should be interpreted with caution because of lack of replication, they suggest that any change in the relative availability of N and P can substantially alter the fungi:bacteria ratio, with bacteria dominating at low N: P ratios as it has been observed previously (Krashevska et al., 2010; Fanin et al., 2013).

Furthermore, the difference in the effect size between litter mass loss and litter SIR after simultaneous N and P additions indicate that the addition of a labile C source during SIR measurement may have also masked a potentially non-negligible C co-limitation on litter decomposition. For instance, the *NP* effect on litter mass loss was the strongest for cellulose (relatively accessible C substrate), intermediate for *G. glabra* or *S. amara* (both litter species relatively rich in DOC), and null for *V. tomentosa* (DOC-poor litter) (**Figure 1**). These results highlight that C availability may modulate the *NP* responses during litter decomposition, confirming that litter C leachates provide the microorganisms with the required energy to efficiently use available nutrients (Cleveland et al., 2006; Fanin et al., 2012). Accordingly, the community structure of microbial decomposers was more dependent on substrate identity than on fertilization treatments (**Table 3**). Cellulose decomposition appears to be mainly driven by fungi, while the leachate C-rich *G. glabra* leaf litter supported the highest proportion of bacteria relative to fungi of all the substrates. Such a C control on microbial community was even stronger in the soil, where substrate identity explained almost three times as much variation in soil SIR (59.4%) than did the fertilization treatments (21.5%) (**Table 2**). The fact that the nature of substrate leachates reaching the soil has a greater impact on soil microbial communities than nutrient fertilization is a rather surprising result (**Figure 5**). However, in line with our finding, Heuck et al. (2015) recently demonstrated that even in P-poor soils, the microbial biomass was primarily limited by C availability, suggesting that P is only secondarily limiting. Collectively, these results suggest that soil microorganisms are strongly limited by easily accessible C compounds originating from fresh litter material rather than by nutrients. C rather than nutrient limitation can in part be a consequence of the better balanced soil C: N: P stoichiometry compared to leaf litter with much wider C: N: P (Fanin et al., 2014; Mooshammer et al., 2014b). Perhaps even more important is the permanent high energy requirement of microorganisms in tropical soils with constantly high temperatures compared to soils at higher latitudes, which may reinforce C limitation in tropical rainforests (Hättenschwiler et al., 2011).

In summary, our study provides clear evidence that shifts in the relative availability of N and P through time regulate the structure and activity of the microbial decomposer community. Collectively, our data suggest that litter mass loss, microbial respiration and microbial community structure can vary temporally along with shifts in the relative availability of nutrients. These changes are rapid and reversible in response to changing nutrient availability. In fact, when both N and P are in excess in the environment, the order in the relative N and P availability did not determine final microbial process rates. The impact of successive N and P additions depended on the identity of decomposing litter material and their nutrient and C status of the soluble fraction, suggesting intimate interactions between exogenous nutrient availability and substrate-specific C and nutrient leachates. Hence, the interactive effects of exogenous N and P availability depend in addition on litter-derived labile C with an even stronger impact on microbial communities in the soil compared to those in the litter layer. The consequences of N and P pulses on microbial communities and their activities in nutrient-poor tropical forests therefore depend to a large extent on litter-specific C quality, and thus, on tree species composition.

## Author Contributions

NiF formulated the idea, NiF and NaF established the analytical methods, conceived and designed the experiment, PS and NiF performed the analyses and collected the data, NiF and NaF analyzed data, NiF, NaF, and SH wrote the manuscript.

## Conflict of Interest Statement

The authors declare that the research was conducted in the absence of any commercial or financial relationships that could be construed as a potential conflict of interest.
