# Supplementary material for: (A)synchronous Availabilities of N and P Regulate the Activity and Structure of the Microbial Decomposer Community
Source: Front Microbiol. 2016 Jan 6;6:1507. doi: 10.3389/fmicb.2015.01507 (PMC4701990; doi:10.3389/fmicb.2015.01507)
Supplement: Supplementary file 1 [file Table_1.DOCX]

**Supplementary 1 | Bacterial and fungal biomass, and F:B ratio in the four different substrates, before (at d_36_) and after (at d_74_) the second nutrient was added.** The percentage between brackets indicates the relative increase or decrease between d_36_ and d_74_.

| Microbial community structure | control | |  | Np | |  | Pn | |  | NP | |
| --- | --- | --- | --- | --- | --- | --- | --- | --- | --- | --- | --- |
|  | d_36_ (+H_2_0) | d_74_ (+H_2_0) |  | d_36_ (+N) | d_74_ (+P) |  | d_36_ (+P) | d_74_ (+N) |  | d_36_ (+NP) | d_74_ (+NP) |
|  |  |  |  |  |  |  |  |  |  |  |  |
| *Cellulose* |  |  |  |  |  |  |  |  |  |  |  |
| Bacterial biomass (µg g^-1^) | 9.8 | 23.8 (+243%) |  | 3.9 | 45.5 (+ 1069%) |  | 12.7 | 10.2 (-20%) |  | 6.4 | 5.8 (-9%) |
| Fungal biomass (µg g^-1^) | 26.1 | 120.5 (+362%) |  | 61.9 | 132.9 (+115%) |  | 37.0 | 46.9 (+27%) |  | 116.0 | 106.2 (-8%) |
| Fungi:bacteria ratio | 2.7 | 5.1 (+90%) |  | 15.9 | 2.9 (-82%) |  | 2.9 | 4.6 (+58%) |  | 18.2 | 18.3 (+0.5%) |
|  |  |  |  |  |  |  |  |  |  |  |  |
| *Goupia glabra* |  |  |  |  |  |  |  |  |  |  |  |
| Bacterial biomass (µg g^-1^) | 14.7 | 23.9 (+62%) |  | 9.7 | 43.3 (+346%) |  | 28.0 | 42.8 (+53%) |  | 33.6 | 38.3 (+14%) |
| Fungal biomass (µg g^-1^) | 28.9 | 27.3 (-5%) |  | 28.9 | 50.5 (+75%) |  | 34.3 | 44.7 (+30%) |  | 43.2 | 38.2 (-11%) |
| Fungi:bacteria ratio | 2.0 | 1.1 (-42%) |  | 3.0 | 1.2 (-61%) |  | 1.2 | 1.0 (-15%) |  | 1.3 | 1.0 (-22%) |
|  |  |  |  |  |  |  |  |  |  |  |  |
| *Simarouba amara* |  |  |  |  |  |  |  |  |  |  |  |
| Bacterial biomass (µg g^-1^) | 12.4 | 10.5 (-15%) |  | 3.2 | 4.1 (+26%) |  | 11.4 | 13.8 (+22%) |  | 4.9 | 6.7 (+36%) |
| Fungal biomass (µg g^-1^) | 26.7 | 16.2 (-39%) |  | 31.8 | 15.1 (-52%) |  | 22.4 | 17.7 (-21%) |  | 29.7 | 22.0 (-26%) |
| Fungi:bacteria ratio | 2.2 | 1.5 (-29%) |  | 9.9 | 3.7 (-62%) |  | 2.0 | 1.3 (-35%) |  | 6.0 | 3.3 (-46%) |
|  |  |  |  |  |  |  |  |  |  |  |  |
| *Vochysia tomentosa* |  |  |  |  |  |  |  |  |  |  |  |
| Bacterial biomass (µg g^-1^) | 9.4 | 13.8 (+48%) |  | 4.9 | 24.2 (+391%) |  | 17.4 | 11.1 (-36%) |  | 18.4 | 17.7 (-4%) |
| Fungal biomass (µg g^-1^) | 38.9 | 22.2 (-43%) |  | 42.4 | 37.1 (-13%) |  | 35.6 | 12.7 (-64%) |  | 36.8 | 15.6 (-58%) |
| Fungi:bacteria ratio | 4.1 | 1.6 (-61%) |  | 8.6 | 1.5 (-82%) |  | 2.0 | 1.1 (-44%) |  | 2.0 | 0.9 (-56%) |
